# Study of Antibiotic Resistance Potential of Spiders Bacteriota and Comparison With the Antibacterial Effect of Essential Oils

**DOI:** 10.1002/mbo3.70145

**Published:** 2025-11-07

**Authors:** Miroslava Kačániová, Joel Horacio Elizondo‐Luevano, Zhaojun Ban, Mária Babošová, Jana Ivanič Porhajašová, Ján Kollár, Przemysław Łukasz Kowalczewski, Stefania Garzoli

**Affiliations:** ^1^ Institute of Horticulture, Faculty of Horticulture and Landscape Engineering Slovak University of Agriculture Nitra Slovakia; ^2^ School of Medical & Health Sciences University of Economics and Human Sciences in Warsaw Warszawa Poland; ^3^ Faculty of Agronomy Universidad Autónoma de Nuevo León (UANL) General Escobedo Nuevo León Mexico; ^4^ School of Biological and Chemical Engineering Zhejiang University of Science and Technology, Zhejiang Provincial Key Laboratory of Chemical and Biological Processing Technology of Farm Products Zhejiang Provincial Collaborative Innovation Center of Agricultural Biological Resources Biochemical Manufacturing Hangzhou China; ^5^ Institute of Plant and Environmental Sciences, Faculty of Agrobiology and Food Resources Slovak University of Agriculture Nitra; ^6^ Institute of Landscape Architecture, Faculty of Horticulture and Landscape Engineering Slovak University of Agriculture Nitra Slovakia; ^7^ Department of Food Technology of Plant Origin Poznań University of Life Sciences Poznań Poland; ^8^ Department of Chemistry and Technologies of Drug Sapienza University Rome Italy

**Keywords:** antibacterial resistance, mass spectrometry, spider microbiota, zoonotic transmission

## Abstract

Insect microorganisms significantly affect the diet, well‐being and behavior of their hosts. However, the microbiota of spiders, which are important natural enemies of pests, is still largely unknown. To gain insight into the bacterial composition of spiders and their possible roles, we collected five different spider species: *Pardosa hortensis*, *Pholcus phalangioides*, *Steatoda bipunctata*, *Steatoda triangulosa* (Walckenaer, 1802), and *Tegenaria domestica* (Clerck, 1757). Mass spectrometry was used to identify the microbiota of each species. After identifying the microbes, the resistence to antibiotics was measured and compared their antibacterial activity with essential oils derived from plants. The antibiotics tested for species‐specific antibacterial activity were imipenem (IPM, 30 mg), ciprofloxacin (CIP, 30 µg/disc), linezolid (LZD, 30 µg/disc), tobramycin (TOB, 30 µg/disc), tigecycline (TGC, 30 µg/disc), and tetracycline (TE, 30 µg/disc). We also tested the antibacterial activity of essential oils from *Cedar atlantica*, *Illicium verum*, and *Pelargonium graveolens*. The most frequently identified species were *Bacillus mycoides* from *P. phalangioides*, *Stutzerimonas chloritidismutans* from *S. bipunctata*, *Aerococcus viridans* and other species from *S. triangulosa*, and *Bacillus mycoides* and *Enterococcus faecium* from *T. domestica*. Antibiotic resistance was present in one‐third of the isolates, with most antibiotic‐resistant bacteria found in *T. domestica*. The essential oils exhibited high antibacterial activity, particularly against *Staphylococcus epidermidis*, *Bacillus pumilus*, *Priestia megaterium*, and *Moraxella osloensis*.

## Graphical abstract

Antibacterial effect comparison between spiders and essential oils.

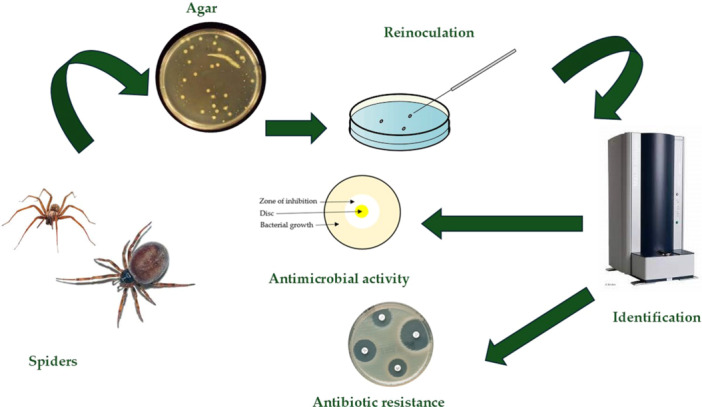

## 1 Introduction

Spider's gut microbial populations are vertically transmissible, and the prey they consume affects the composition of these communities (Kennedy et al. 2020; Sheffer et al. 2019). These microbes might provide energy and help the body combat parasites and infections (Moran et al. 2019). Due to its ability to influence digestion, metabolism, and health maintenance, the microbiota of spiders can be crucial to their survival (Kumar 2020). Numerous characteristics of spiders are influenced by their gut microbiome, and new research has revealed that a wide variety of endosymbionts live in and on spiders (Zhang et al. 2018). Additionally, it has been demonstrated that while spiders on different webs have more noticeable variations in their microbial compositions, those on the same web have relatively similar levels of microbial richness (Busck et al. 2020).

Animals that live in human habitats, or synanthropic animals, can serve as important carriers and reservoirs of harmful microorganisms. Bacteria can colonize wild, domesticated, and captive animals, and they can serve as reservoirs for the spread of infections through physical contact, such as bites, stings, and scratches. A wide variety of synanthropic niches are occupied by spiders (Nentwig 2015). They can catch and eat a wide variety of prey, including fish, lizards, snakes, birds, rodents, big arthropods, and medically significant pests like house flies and mosquitoes (Barin et al. 2010; Eljounaidi et al. 2016; Wang et al. 2011). Some people are willing to eat carrion (Barrantes and Weng 2007; Sandidge 2003; Vetter 2011). In laboratory settings, we saw wild‐caught species, such as *Steatoda nobilis*, feeding on dead food for up to 8 days (Dunbar et al. 2020). While arthropods' innate immune systems guard against harmful microorganisms (Baxter et al. 2017; Kavanagh and Reeves 2007; Savitzky et al. 2012), the bacteria are free to proliferate and flourish on their host's corpse after death. Microbes will inevitably come into contact with spiders through their surroundings or food, particularly carrion. Spiders have been linked to bite incidents that resulted in bacterial infections28, and they may thus harbor dangerous bacteria (Ahrens 2011; Monteiro et al. 2002).

The growing worldwide health concern of antimicrobial resistance (AMR) poses a danger to the effectiveness of antibiotics, which have long been essential to contemporary medicine (Bogri et al. 2024). The emergence of AMR has often been linked to the direct use of antibiotics. However, a growing body of research indicates that non‐pathogenic (symbiotic) microbiome bacteria play a crucial role in the transmission of resistance genes within complex communities and in their propagation (Bottery et al. 2021; Tams et al. 2023). Antimicrobial resistance genes (ARGs) can be stored in the microbiome by bacteria, and the possibility of horizontal gene transfer between these and pathogenic bacteria can result in the formation of multidrug‐resistant infections, exacerbating the AMR issue (Holmes et al. 2016). Since the advent of antibiotics (ATBs), the field of medicine has undergone a transformative period, significantly improving human health and longevity. However, the widespread use of antibiotics has led to a global health crisis. AMR has become an increasingly critical issue over time (Singh et al. 2003).

As an alternative to synthetic medications for the treatment of bacterial infections, a variety of aromatic and therapeutic plants, herbs, and spices have been suggested as a major source of natural antimicrobials (Puvača et al. 2020). Because of the high concentration of bioactive chemicals in medicinal plants, its essential oil has been employed extensively for this purpose (Canter et al. 2005; Cosentino et al. 1999; Joana Gil‐Chávez et al. 2013). Essential oils have demonstrated efficacy in treating intestinal problems (Tanveer et al. 2020), respiratory disorders (Ali et al. 2005), infectious diseases of the urinary tract (Ebani et al. 2018), and diseases of the skin (Abu‐Al‐Basal 2010). Aromatic and oily liquids possessing antibacterial, antifungal, antioxidant, and anti‐inflammatory properties are termed essential oils (EOs). These oils are extracted from plants (Bocate et al. 2021; Evangelista et al. 2021; Güths et al. 2022).

The essential oil of *Cedrus atlantica* (CAEO) has a variety of antibacterial properties against various microorganisms. *Salmonella enterica*, a gram‐negative bacterium, was found to be less sensitive to CAEO than *Staphylococcus aureus* and *Micrococcus luteus* (Kačániová et al. 2022b). The antibacterial activity of CAEO against *Escherichia coli, Bacillus subtilis, M. luteus*, and *S. aureus* was also determined by Satrani et al. (2006). The most effective EO for *E. coli* strains was reported to be *Illicum verum* (IV) by Freire et al. (2011). Noumi et al. (2023) did, however, also demonstrate that IVEO was more effective against Gram‐positive bacteria (*Staphylococcus aureus*) than Gram‐negative bacteria (*Pseudomonas aeruginosa, Shigella flexeneri*, and *Vibrio vulnificus*). This oil showns more efficacy against *S. aureus* than *E. coli* in other studies (Damayanti). IVEO is also highly effective against G^+^ bacteria, including methicillin‐resistant *S. aureus* (MRSA), according to earlier research (Muhsinah et al. 2022). Additionally, a qualitative assessment of IVEO's susceptibility testing capability was carried out with a set of clinical isolates that are multidrug‐resistant (MDR), including *Streptococcus pneumoniae, S. aureus, Klebsiella pneumoniae, E. coli, Acinetobacter baumannii*, and *P. aeruginosa* (Semeniuc et al. 2017). A number of microorganisms have shown encouraging responses to the antibacterial properties of *Pelargonium graveolans* (PG) essential oil (Khokra et al. 2008; Lis‐Balchin et al. 1998). The reported antibacterial qualities of PGEO indicate that they have a good chance of being used as natural preservatives and antimicrobial agents in various products.

In contrast to molecular identification methods and biochemical‐based tests, MALDI‐TOF MS (matrix‐assisted laser desorption ionization–time of flight mass spectrometry) has gained popularity as a speedy, affordable, and labor‐efficient method for microbiological identification in clinical settings. Either the PMF (Protein Mass Fingerprint) of the measured microorganism is compared to PMF databases, or the masses of the detected biomarkers of unknown species are matched using proteomic databases to conduct identification based on MALDI‐TOF MS measurements (Singhal et al. 2015). Because Uchida‐Fujii et al. (2020) were able to identify 86.2% of 3724 isolates at the species level, they demonstrated the potential of MALDI‐TOF MS (Bruker Biotyper) in environmental microbiology.

The aim of this study was to investigate the presence of an exogenous bacteriota with MALDI‐TOF MS Biotyper in spiders and to test its antibacterial resistance to antibiotics and plant essential oils. Many bacteria, including exogenous bacteriota of spiders, can exhibit antibiotic resistance and therefore our no less important aim was to investigate the antibacterial activity of plant essential oils against spider bacteria.

## 2 Materials and Methods

### 2.1 Sample Collection

The samples were collected at the Slovak Agricultural University in Nitra (48°18'N 18°05E), Slovak Republic. The spiders were collected from the buildings of the University. Our study involved a total of one hundred male spiders from the following species: *Pardosa hortensis* with 20 samples, *Pholcus phalangioides* with 20 samples, *Steatoda bipunctata* with 20 samples, *Steatoda triangulosa* (Walckenaer, 1802) with 20 samples, and *Tegenaria domestica* (Clerck, 1757) with 20 samples. The spiders were identified using microscopy. All species were confirmed to be non‐endangered and non‐protected. Upon collection, each spider was frozen at −20°C for 1 min. Subsequently, each spider was placed into a sterile 2 mL microcentrifuge tube, and 1 mL of sterile 0.87% (w/v) NaCl solution was added to sample the exterior surfaces of the spiders. Following this, 100 µL of the sample was plated onto agar plates for bacterial identification.

### 2.2 Microbiological Analyses

The total microbial count, Enterobacteriales, anaerobic, and fastidious microorganisms were identified using the following agar media in sequence: Tryptone Soya Agar (TSA), Tryptone Sugar Iron Agar (TSI), Anaerobic Agar (AA), and Blood Agar (BA) supplemented with 7% horse blood (Sigma‐Aldrich, St. Louis, MO, USA). TSA plates were incubated for 24–48 h at 30°C, TSI agar for 18–24 h at 37°C, AA for 24–48 h at 30°C under anaerobic conditions, and BA for 24–48 h at 37°C aerobically. After incubation, eight bacterial colonies with distinct macroscopic features were selected from each agar type for species confirmation. Isolates chosen for MALDI‐TOF identification were subcultured on TSA plates and further incubated at 37°C for 24 h.

### 2.3 Species Identification

The microbiota was identified using a MALDI‐TOF MS Biotyper (Bruker Daltonics, Bremen, Germany). Samples were prepared following the manufacturer's instructions for the MALDI TOF MS Biotyper. Initially, the bacterial suspension was centrifuged for 2 min at 14,000 rpm in 300 µL of distilled water and 900 µL. After removing the supernatant, another centrifugation step was performed with the addition of 10 µL of 70% formic acid and 10 µL of acetonitrile to the pellet, followed by centrifugation for 2 min at 14,000 rpm Subsequently, 1 μL of the supernatant was used for analysis, and the suspension was coated with 1 μL of a matrix containing α‐Cyano‐4‐hydroxycinnamic acid. For identification, the Microflex LT instrument (Bruker Daltonics, Bremen, Germany) and Flex Control 3.4 software were utilized, alongside BC‐specific software for Biotyper Realtime Classification 3.1. Confidence values of ≥ 2.0 and ≥ 1.7 were employed for species and genus level identifications, respectively (Kačániová et al. 2022b).

### 2.4 Antibiotic Resistance

Antibiotic susceptibility tests were conducted using the disc diffusion method. The EUCAST guidelines (EUCAST, 2024), were followed to assess antibiotic resistance for each microbiological species isolated from spiders. The following Oxoid antibiotic discs were used: imipenem (IPM, 30 µg), ciprofloxacin (CIP, 30 µg), linezolid (LZD, 30 µg), tobramycin (TOB, 30 µg), tigecycline (TGC, 30 µg), and tetracycline (TE, 30 µg). Antibacterial resistance testing followed EUCAST protocols ((EUCAST 2004).

Bacterial isolates were cultured in Mueller‐Hinton broth (Sigma‐Aldrich, St. Louis, MO, USA) for 24 h at 37°C. Following this, microbial suspensions in sterile distilled water were adjusted to a concentration of 10^8^ cells/mL (equivalent to McFarland standard 0.5). Each isolated strain underwent testing three times.

### 2.5 Antibacterial Activity

To assess the antibacterial activity of the essential oils (EOs), the disc diffusion method was employed. Bacterial inoculum with an optical density corresponding to 0.5 McFarland standard (1.5 × 10^8^ CFU/mL) was prepared by adjusting a 24‐h culture in Mueller Hinton broth (Oxoid, Basingstoke, UK) at 37°C. Subsequently, the bacteria were spread on Mueller Hinton agar plates. Sterile 6 mm discs soaked with 10 µL of each EO suspension were then placed on the agar surface and incubated for an additional 24 h at 37°C. Analyses were conducted in triplicate. Three essential oils were used for the antibacterial activity assessment. The *Cedar atlantica* EO (CAEO), provided by Hanus s.r.o. (Nitra, Slovakia), was hydrodistilled from cedar wood originating from Morocco. It was stored at 4°C in darkness throughout the study, and its chemical composition was previously reported by (Kačániová et al. 2022a). The second EO, obtained from Hanus s.r.o. (Nitra, Slovakia), was distilled from dried *Illicium verum* (IVEO) fruits imported from China. The fruits were stored in the dark conditions at 4°C before analysis, and their chemical composition was evaluated by Kačániová et al. (2024a). The third EO used in the study was extracted by steam distillation from fresh *Pelargonium graveolens* (PGEO) wort sourced from Egypt. Similar to the others, it was stored at 4°C in darkness during the examination, and its chemical composition was previously published by (Kačániová et al. 2023b).

### 2.6 Statistical Evaluation

For statistical analysis, we utilized the One‐way Analysis of Variance (ANOVA) to assess whether significant differences existed between groups. Subsequently, Tukey's Honest Significant Difference (HSD) test was applied to determine specific group differences following the ANOVA. The significance level was set at *p*≤ 0.05. All statistical analyses were conducted using the online Astatsa ANOVA One Way tool. This rigorous approach enabled us to thoroughly evaluate differences among multiple groups and ensure the reliability of our findings.

## 3 Results

### 3.1 Microbiological Analyses

The bacterial counts are detailed in Table 1. On Anaerobic agar, counts ranged from 1.26 log CFU/g in the spider species *P. phalangioides* and *S. triangulosa* to 1.53 log CFU/g in *P. hortensis*. The total bacterial count on Blood agar varied from 1.22 log CFU/g in *P. phalangioides* to 1.86 log CFU/g in *P. hortensis*, while on Triple Sugar Iron agar, counts ranged from 1.21 log CFU/g in *P. phalangioides* to 2.02 log CFU/g in *P. hortensis*. Bacterial counts on Tryptone Soya agar ranged from 1.22 log CFU/g in *P. phalangioides* to 1.64 log CFU/g in *P. hortensis*.

**Table 1:** Counts of microorganisms (log CFU/g) of the spiders.

Sample	AA log CFU/g	BA log CFU/g	TSA log CFU/g	TSI log CFU/g
*Pardosa hortensis*	1.53 ± 0.14^a^	1.86 ± 0.14^a^	2.02 ± 0.21^a^	1.64 ± 0.15^a^
*Pholcus phalangioides*	1.26 ± 0.08^b^	1.22 ± 0.10^b^	1.21 ± 0.08^b^	1.22 ± 0.06^b^
*Steatoda bipunctata*	1.37 ± 0.14^b^	1.49 ± 0.33^c^	1.58 ± 0.42^c^	1.40 ± 0.20^c^
*Steatoda triangulosa*	1.26 ± 0.08^b^	1.40 ± 0.23^b,c^	1.35 ± 0.19^b,c^	1.32 ± 0.14^b,c^
*Tegenaria domestica*	1.36 ± 0.15^b^	1.27 ± 0.14^b^	1.27 ± 0.15^b^	1.25 ± 0.14^b,c^

*Note:* Values marked with the same letter in the columns are not significantly different *p* > 0.05 (One‐way ANOVA Tukey test).

Abbreviations: AA, anaerobic agar; BA, blood agar; TSA, tryptone soya agar; TSI, triple sugar iron agar.

**Figure 1:**
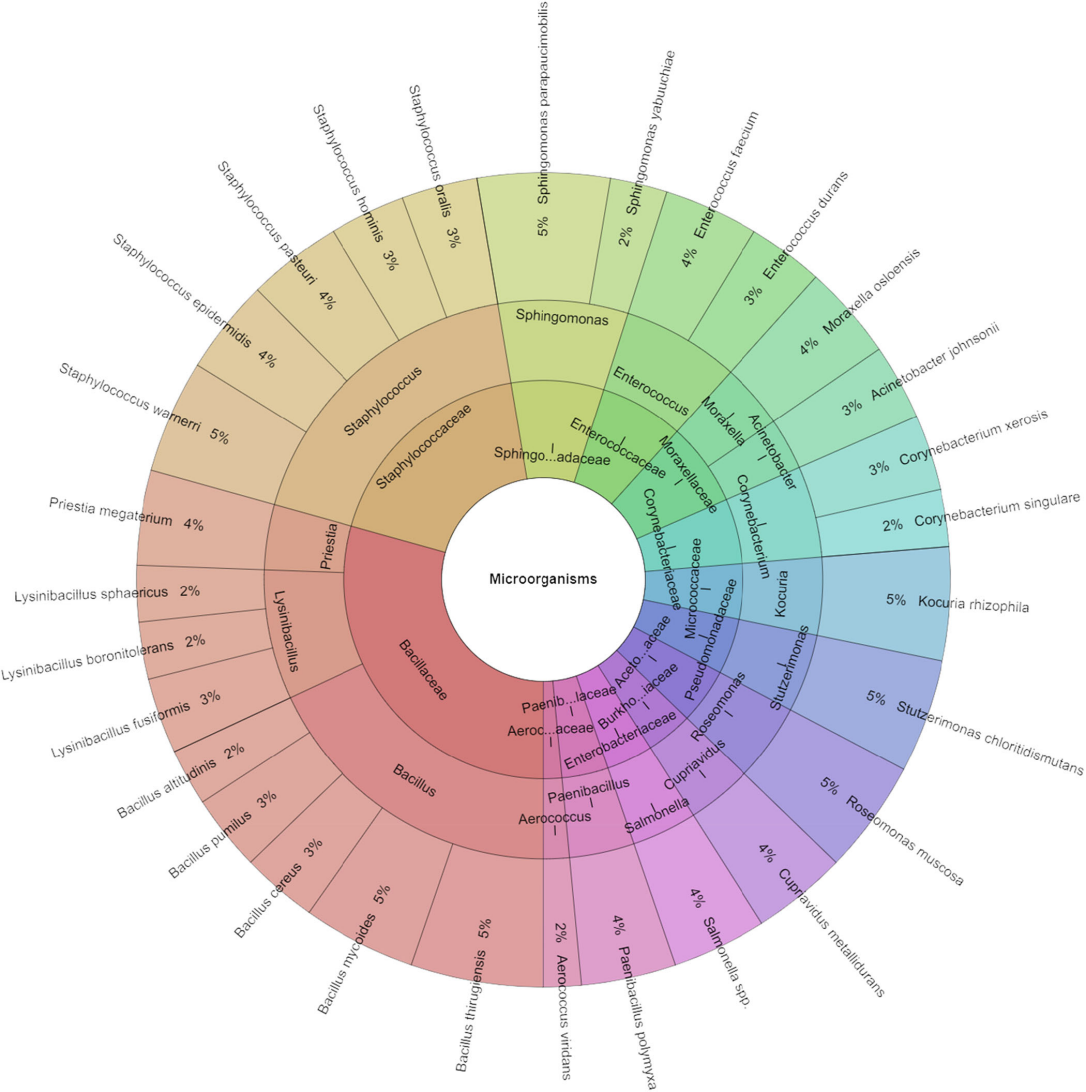
Krona chart: The microbiota of *Pardosa hortensis*.

Figure 1 displays the isolated species of bacteria from *P. hortensis*. A total of 29 species, 16 genera, and 13 families were isolated from *P. hortensis*. The highest number of isolates, totaling 133, were obtained. The most frequently isolated species included *Bacillus mycoides*, *Bacillus thurgiensis*, *Sphingomonas paucimobilis*, *Staphylococcus warneri*, and *Stutzerimonas chloritidismutans*, each comprising 5% of the isolates.

**Figure 2:**
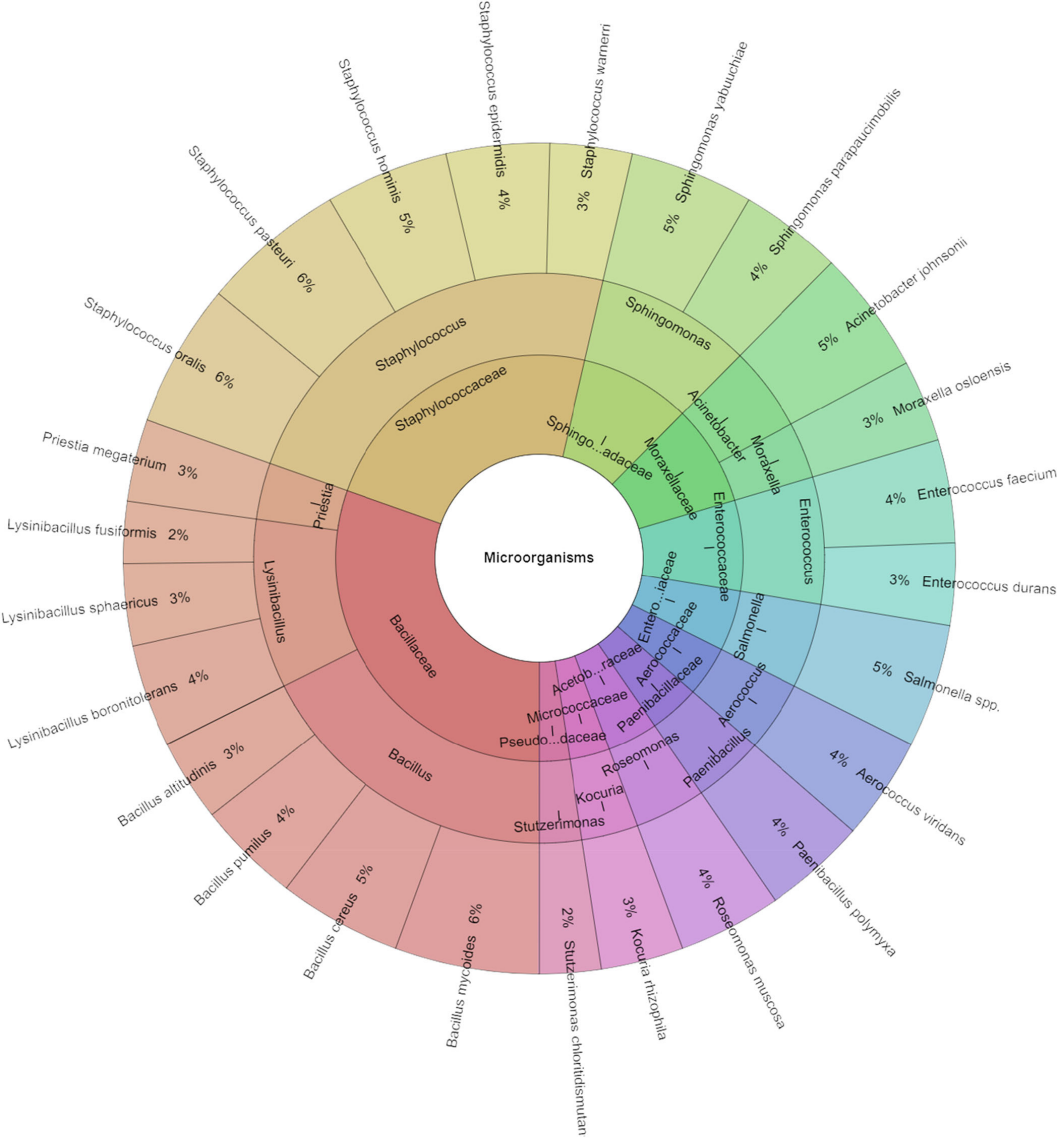
Krona chart: The microbiota of *Pholcus phalangioides*.

Figure 2 shows bacteria isolated from *Pholcus phalangioides* spiders, a total of 125 isolates with a score above 2. In total, 12 families, 14 genera and 25 species were identified. The most frequently isolated species from the spider *P. phalangioides* were *Bacillus mycoides*, *Staphylococcus oralis* and *Staphylococcus pasteuri*, each accounting for 6% of the isolates.

**Figure 3:**
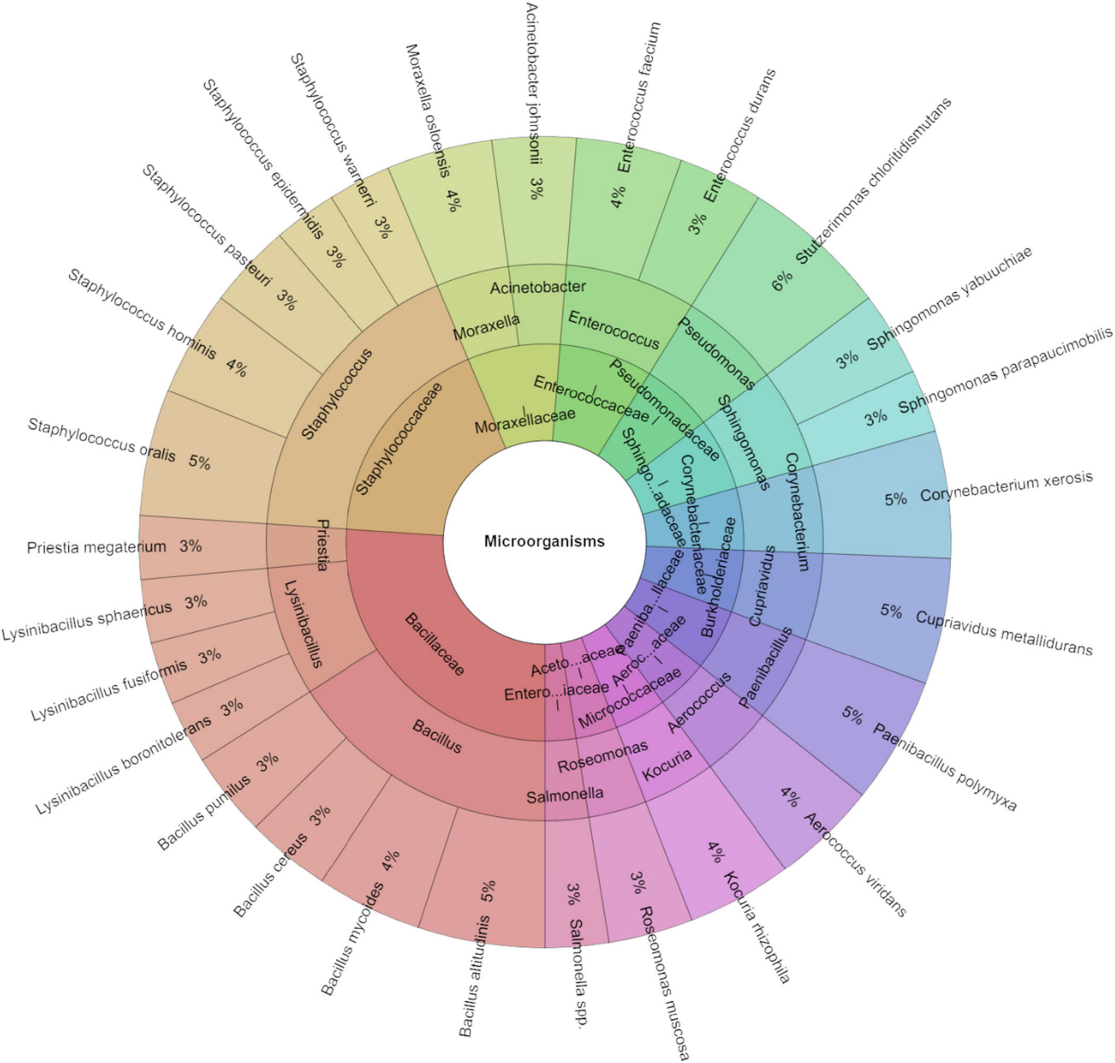
Krona chart: The microbiota of *Steatoda bipunctata*.

Figure 3 displays the bacteria isolated from the exogenous microbiota of *Steatoda bipunctata* spiders, totaling 119 isolates with a score higher than 2. The most frequently isolated species from *Steatoda bipunctata* was *Stutzerimonas chloritidismutans* at 6%, followed by *Bacillus altitudinis*, *Corynebacterium xerosis*, *Cupriavidus metallidurans*, and *Staphylococcus oralis*, each at 5%. In total, 27 species, 16 genera, and 13 families were identified within this group of spiders.

**Figure 4:**
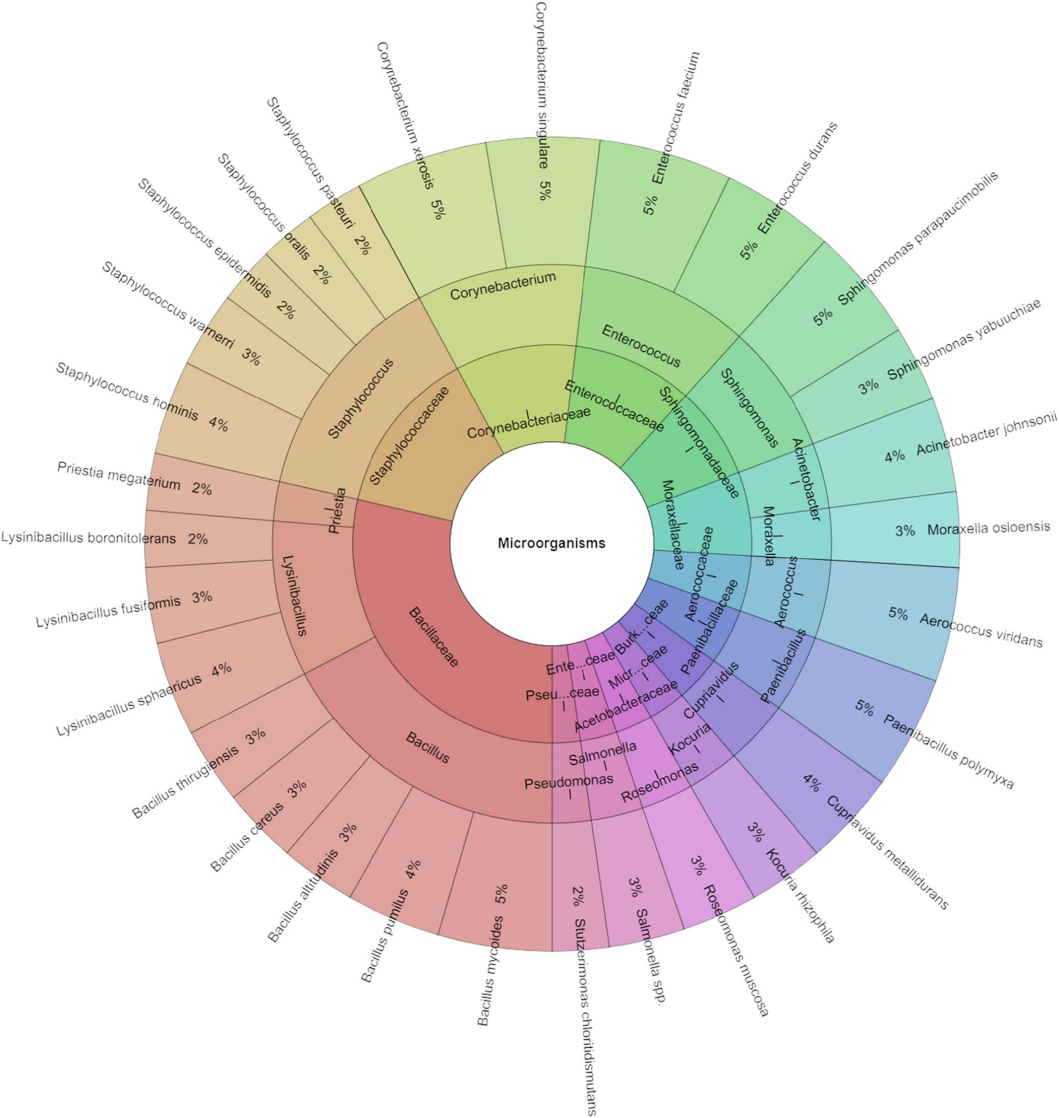
Krona chart: The microbiota of *Steatoda triangulosa*.

The composition of the microbiota of the arthropod *Steatoda triangulosa* is shown in Figure 4. A total of 133 isolates were identified in this spider group. Twenty‐nine species, 16 genera and 13 families were isolated, with *Aerococcus viridans*, *Corynebacterium singulare*, *Corynebacterium xerosis*, *Enterococcus durans*, *Enterococcus faecium* and *Sphingomonas parapaucimobilis* having the highest number of isolates with 5%.

**Figure 5:**
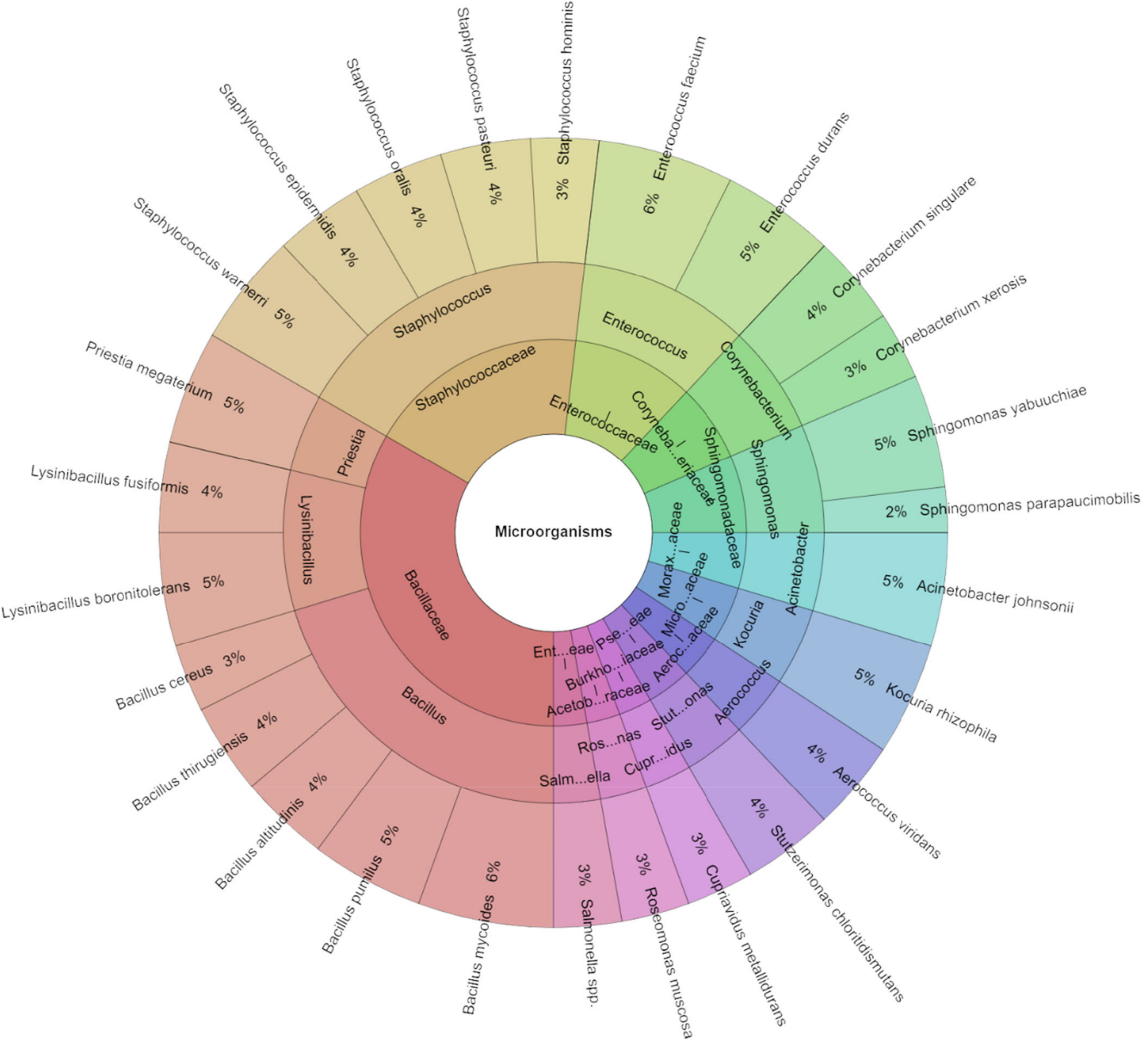
Krona chart: The microbiota of *Tegenaria domestica*.

Figure 5 depicts the microbiota of *Tegenaria domestica* arthropods. A total of 108 isolates were identified, belonging to 12 families, 14 genera, and 26 species. The most frequently isolated species were *Bacillus mycoides* and *Enterococcus faecium*, each at 6%, followed by *Acinetobacter johnsonii*, *Paenibacillus megaterium*, *Bacillus pumilus*, *Enterococcus durans*, *Kocuria rhizophila*, *Lysinibacillus boronitolerans*, *Sphingomonas yabuuchiae*, and *Staphylococcus warneri*, each at 5%.

### 3.2 Antibiotic Resistance of Isolated Microorganisms and Their Sensitivity to EOs

Table 2 shows the antibiotic resistance of bacteria isolated from *Pardosa hortensis*. Ninety‐three different species were found to be resistant to different antibiotics after isolation from *Pardosa hortensis*. Susceptibility to antibiotic resistance was found in 151 isolates. The antibacterial activity of CAEO ranged from 5.67 to 12.33 mm. CAEO was most effective against *Moraxella osloensis* and *Stapylococcus warnerri*. The best antibacterial activity of IVEO was found against *Moraxella osloensis* and PGEO against *Moraxella osloensis* and *Stapylococcus epidermidis* (12.33 mm).

**Table 2:** Antibacterial resistance and activity of EOs against bacteria isolated from *Pardosa hortensis*.

	Antibiotic resistance R/S			Antibacterial activity in mm		
Isolated bacteria	IMP	CIP	TOB	CAEO	IVEO	PGEO
*Acinetobacter johnsonii*	1/3	2/2	4/0	6.33 ± 0.58	7.33 ± 0.58	6.33 ± 0.58
*Aerococcus viridans*	ND	ND	ND	6.67 ± 0.58	5.67 ± 0.58	6.67 ± 0.58
	**IMP**	**CIP**	**LZD**	**CAEO**	**IVEO**	**PGEO**
*Bacillus altitudinis*	1/2	0/3	3/0	7.67 ± 0.58	6.33 ± 0.58	8.67 ± 0.58
*Bacillus cereus*	2/2	1/3	0/4	10.33 ± 0.58	8.67 ± 0.58	8.33 ± 0.58
*Priestia megaterium*	2/3	1/4	0/5	6,67 ± 0.58	8.33 ± 0.58	9.67 ± 0.58
*Bacillus mycoides*	2/4	3/3	1/5	4.67 ± 0.58	7.33 ± 0.58	8.67 ± 0.58
*Bacillus pumilus*	2/2	1/3	0/4	7.33 ± 0.58	6.67 ± 0.58	9.33 ± 0.58
*Bacillus thirugiensis*	3/4	4/3	5/2	6.67 ± 0.58	3,67 ± 0.58	6.33 ± 0.58
	**CIP**	**TET**	**LZD**	**CAEO**	**IVEO**	**PGEO**
*Corynebacterium singulare*	0/3	1/2	1/2	7.67 ± 0.58	8.33 ± 0.58	11,33 ± 0.58
*Corynebacterium xerosis*	2/2	1/3	3/1	7.33 ± 0.58	6.67 ± 0.58	5.67 ± 0.58
*Cupriavidus metallidurans*	ND	ND	ND	4.33 ± 0.58	4.67 ± 0.58	5.67 ± 0.58
	**CIP**	**TGC**	**LZD**	**CAEO**	**IVEO**	**PGEO**
*Enterococcus durans*	1/3	2/2	3/1	9.33 ± 0.58	7.67 ± 0.58	10.67 ± 0.58
*Enterococcus faecium*	2/3	1/4	3/2	7.67 ± 0.58	8.33 ± 0.58	7.67 ± 0.58
*Kocuria rhizophila*	ND	ND	ND	8.67 ± 0.58	7.67 ± 0.58	8.67 ± 0.58
	**IMP**	**CIP**	**LZD**	**CAEO**	**IVEO**	**PGEO**
*Lysinibacillus boronitolerans*	ND	ND	ND	6.67 ± 0.58	4.33 ± 0.58	5.33 ± 0.58
*Lysinibacillus fusiformis*	ND	ND	ND	7.33 ± 0.58	6.67 ± 0.58	7.33 ± 0.58
*Lysinibacillus sphaericus*	ND	ND	ND	4.67 ± 0.58	5.33 ± 0.58	6.33 ± 0.58
*Moraxella osloensis*	ND	ND	ND	12.33 ± 0.58	11.67 ± 0.58	12.33 ± 0.58
*Paenibacillus polymyxa*	ND	ND	ND	11.67 ± 0.58	10.67 ± 0.58	9.33 ± 0.58
*Stutzerimonas chloritidismutans*	ND	ND	ND	6.67 ± 0.58	7.33 ± 0.58	4.67 ± 0.58
*Roseomonas muscosa*	ND	ND	ND	8.67 ± 0.58	8.33 ± 0.58	9.67 ± 0.58
	**IMP**	**TOB**	**TGC**	**CAEO**	**IVEO**	**PGEO**
*Salmonella* spp.	1/4	3/2	2/3	5.67 ± 0.58	7.33 ± 0.58	8.33 ± 0.58
*Sphingomonas parapaucimobilis*	ND	ND	ND	6.67 ± 0.58	5.33 ± 0.58	6.33 ± 0.58
*Sphingomonas yabuuchiae*	ND	ND	ND	7.33 ± 0.58	7.67 ± 0.58	8.33 ± 0.58
	**TOB**	**TGC**	**LZD**	**CAEO**	**IVEO**	**PGEO**
*Staphylococcus epidermidis*	1/4	2/3	3/2	9.67 ± 0.58	11.33 ± 0.58	12.33 ± 0.58
*Staphylococcus hominis*	1/3	2/2	1/3	8.67 ± 0.58	9.33 ± 0.58	9.67 ± 0.58
*Staphylococcus oralis*	1/3	2/2	3/1	8.67 ± 0.58	9.67 ± 0.58	8.67 ± 0.58
*Staphylococcus pasteuri*	3/2	1/4	0/5	7.67 ± 0.58	8.67 ± 0.58	9.33 ± 0.58
*Staphylococcus warnerri*	4/2	2/4	3/3	12.33 ± 0.58	8.67 ± 0.58	9.67 ± 0.58
**Total R/S**	**29/49**	**29/50**	**35/52**			

Abbreviations: CAEO, *Cedar atlantica* essential oil; CIP, ciprofloxacin; IPM, imipenem; IVEO, *Illicium verum* essential oil; LZD, linezolid; ND, not detected; PGEO, *Pelargonium graveolens* essential oil; TE, tetracycline; TGC, tigecycline; TOB, tobramycin;

The antibiotic resistance of bacteria isolated from *Pholcus phalangioides* is shown in Table 3. Ninety‐three different species isolated from *Pholcus phalangioides* were found to be resistant to different antibiotics. Antibiotic susceptibility to resistance was found in 144 isolates. The range of antibacterial activity of CAEO was 2.33 to 12.33 mm. CAEO was most effective against *Acinetobacter johnsnii*, *Bacillus altitudinis* and *Moraxella osloensis*. IVEO and PGEO showed the strongest antibacterial activity against *Moraxella osloensis* (12.33 mm).

**Table 3:** Antibiotic resistance and antibacterial activity of EOs against bacteria isolated from *Pholcus phalangioides*.

	Antibiotic resistance R/S			Antibacterial activity in mm		
Isolated bacteria	IMP	CIP	TOB	CAEO	IVEO	PGEO
*Acinetobacter johnsonii*	2/4	3/3	1/5	12.33 ± 0.58	11.67 ± 0.58	9.67 ± 0.58
*Aerococcus viridans*	ND	ND	ND	9.33 ± 0.58	6.67 ± 0.58	8.67 ± 0.58
	**IMP**	**CIP**	**LZD**	**CAEO**	**IVEO**	**PGEO**
*Bacillus altitudinis*	1/3	2/2	0/4	12.33 ± 0.58	9.67 ± 0.58	11.33 ± 0.58
*Bacillus cereus*	0/6	3/3	2/4	9.67 ± 0.58	8.67 ± 0.58	9.33 ± 0.58
*Priestia megaterium*	2/2	1/3	0/4	8.67 ± 0.58	8.67 ± 0.58	9.67 ± 0.58
*Bacillus mycoides*	5/2	3/4	2/5	9.33 ± 0.58	7.67 ± 0.58	8.67 ± 0.58
*Bacillus pumilus*	3/2	2/3	1/4	9.67 ± 0.58	12.33 ± 0.58	8.67 ± 0.58
	**CIP**	**TGC**	**LZD**	**CAEO**	**IVEO**	**PGEO**
*Enterococcus durans*	1/3	2/2	3/1	9.33 ± 0.58	7.67 ± 0.58	10.67 ± 0.58
*Enterococcus faecium*	2/3	3/2	4/1	7.67 ± 0.58	8.33 ± 0.58	7.67 ± 0.58
*Kocuria rhizophila*	ND	ND	ND	8.67 ± 0.58	7.67 ± 0.58	8.67 ± 0.58
	**IMP**	**CIP**	**LZD**	**CAEO**	**IVEO**	**PGEO**
*Lysinibacillus boronitolerans*	ND	ND	ND	5.33 ± 0.58	6.67 ± 0.58	6.67 ± 0.58
*Lysinibacillus fusiformis*	ND	ND	ND	7.33 ± 0.58	7.33 ± 0.58	7.33 ± 0.58
*Lysinibacillus sphaericus*	ND	ND	ND	6.33 ± 0.58	4.67 ± 0.58	4.67 ± 0.58
*Moraxella osloensis*	ND	ND	ND	12.33 ± 0.58	12.33 ± 0.58	12.33 ± 0.58
*Paenibacillus polymyxa*	ND	ND	ND	9.33 ± 0.58	11.67 ± 0.58	11.67 ± 0.58
*Stutzerimonas chloritidismutans*	ND	ND	ND	4.67 ± 0.58	6.67 ± 0.58	6.67 ± 0.58
*Roseomonas muscosa*	ND	ND	ND	9.67 ± 0.58	8.67 ± 0.58	8.67 ± 0.58
	**IMP**	**TOB**	**TGC**	**CAEO**	**IVEO**	**PGEO**
*Salmonella* spp.	2/4	1/5	3/3	5.33 ± 0.58	6.67 ± 0.58	5.67 ± 0.58
*Sphingomonas parapaucimobilis*	ND	ND	ND	7.67 ± 0.58	8.33 ± 0.58	7.67 ± 0.58
*Sphingomonas yabuuchiae*	ND	ND	ND	4.67 ± 0.58	6.33 ± 0.58	5.67 ± 0.58
	**TOB**	**TGC**	**LZD**	**CAEO**	**IVEO**	**PGEO**
*Staphylococcus epidermidis*	2/3	3/2	1/4	7.67 ± 0.58	8.33 ± 0.58	6.33 ± 0.58
*Staphylococcus hominis*	3/3	1/5	2/4	8.33 ± 0.58	7.33 ± 0.58	7.67 ± 0.58
*Staphylococcus oralis*	0/7	1/6	7/0	5.33 ± 0.58	4.33 ± 0.58	7.67 ± 0.58
*Staphylococcus pasteuri*	5/2	3/4	4/3	2.33 ± 0.58	3.67 ± 0.58	4.33 ± 0.58
*Staphylococcus warnerri*	2/2	4/0	1/3	5.67 ± 0.58	6.33 ± 0.58	6.67 ± 0.58
**Total R/S**	**30/46**	**32/44**	**31/54**			

Abbreviations: CCAEO, *Cedar atlantica* essential oil; CIP, ciprofloxacin; IPM, imipenem; IVEO, *Illicium verum* essential oil; LZD, linezolid; ND, not detected; PGEO, *Pelargonium graveolens* essential oil; TOB, tobramycin; TGC, tigecycline; TE, tetracycline.

Table 4 presents the antibiotic resistance and antibacterial activity of essential oils against bacteria isolated from *Steatoda bipunctata*. A total of 64 species isolated from *Steatoda bipunctata* exhibited resistance to various antibiotics, while 131 isolates showed sensitivity to antibiotic treatments. The antibacterial activity of CAEO ranged from 3.67 to 12.33 mm, IVEO from 4.67 to 12.33 mm, and PGEO from 3.67 to 13.67 mm. CAEO was most effective against *S. epidermidis*, IVEO against *Staphylococcus hominis*, and PGEO against *S. epidermidis*.

**Table 4:** Antibiotic resistance and antibacterial activity of EOs against bacteria isolated from *Steatoda bipunctata*.

	Antibiotic resistance R/S			Antibacterial activity in mm		
Isolated bacteria	IMP	CIP	TOB	CAEO	IVEO	PGEO
*Acinetobacter johnsonii*	1/3	2/2	4/0	5.67 ± 0.58	6.33 ± 0.58	7.67 ± 0.58
*Aerococcus viridans*	ND	ND	ND	6.33 ± 0.58	6.67 ± 0.58	5.33 ± 0.58
	**IMP**	**CIP**	**LZD**	**CAEO**	**IVEO**	**PGEO**
*Bacillus altitudinis*	0/6	0/6	2/4	7.67 ± 0.58	8.33 ± 0.58	6.67 ± 0.58
*Bacillus cereus*	1/3	2/2	3/1	9.67 ± 0.58	10.33 ± 0.58	8.67 ± 0.58
*Priestia megaterium*	0/3	1/2	0/3	9.33 ± 0.58	11.67 ± 0.58	12.33 ± 0.58
*Bacillus mycoides*	2/3	3/2	0/5	10.33 ± 0.58	7.67 ± 0.58	8.67 ± 0.58
*Bacillus pumilus*	1/3	2/2	0/4	8.33 ± 0.58	7.67 ± 0.58	9.33 ± 0.58
	**CIP**	**TET**	**LZD**	**CAEO**	**IVEO**	**PGEO**
*Corynebacterium xerosis*	0/6	3/3	2/4	9.33 ± 0.58	8.33 ± 0.58	6.67 ± 0.58
*Cupriavidus metallidurans*	ND	ND	ND	8.67 ± 0.58	7.33 ± 0.58	8.33 ± 0.58
	**CIP**	**TGC**	**LZD**	**CAEO**	**IVEO**	**PGEO**
*Enterococcus durans*	2/2	1/3	0/4	4.33 ± 0.58	5.67 ± 0.58	4.67 ± 0.58
*Enterococcus faecium*	2/3	1/4	3/2	6.33 ± 0.58	6.33 ± 0.58	7.33 ± 0.58
*Kocuria rhizophila*	ND	ND	ND	4.67 ± 0.58	5.33 ± 0.58	3.67 ± 0.58
	**IMP**	**CIP**	**LZD**	**CAEO**	**IVEO**	**PGEO**
*Lysinibacillus boronitolerans*	ND	ND	ND	4.67 ± 0.58	7.33 ± 0.58	4.67 ± 0.58
*Lysinibacillus fusiformis*	ND	ND	ND	7.33 ± 0.58	6.67 ± 0.58	7.67 ± 0.58
*Lysinibacillus sphaericus*	ND	ND	ND	3.67 ± 0.58	2.33 ± 0.58	3.67 ± 0.58
*Moraxella osloensis*	ND	ND	ND	4.33 ± 0.58	4.67 ± 0.58	5.67 ± 0.58
*Paenibacillus polymyxa*	ND	ND	ND	6.33 ± 0.58	4.67 ± 0.58	5.33 ± 0.58
*Stutzerimonas chloritidismutans*	ND	ND	ND	4.67 ± 0.58	5.67 ± 0.58	4.56 ± 0.58
*Roseomonas muscosa*	ND	ND	ND	6.67 ± 0.58	7.33 ± 0.58	8.33 ± 0.58
	**IMP**	**TOB**	**TGC**	**CAEO**	**IVEO**	**PGEO**
*Salmonella* spp.	0/3	3/0	1/2	7.67 ± 0.58	8.33 ± 0.58	9.33 ± 0.58
*Sphingomonas parapaucimobilis*	ND	ND	ND	9.67 ± 0.58	8.67 ± 0.58	6.67 ± 0.58
*Sphingomonas yabuuchiae*	ND	ND	ND	8.33 ± 0.58	9.33 ± 0.58	7.67 ± 0.58
	**TOB**	**TGC**	**LZD**	**CAEO**	**IVEO**	**PGEO**
*Staphylococcus epidermidis*	1/2	2/1	2/1	12.33 ± 0.58	11.67 ± 0.58	13.67 ± 0.58
*Staphylococcus hominis*	2/3	1/4	0/5	11.67 ± 0.58	12.33 ± 0.58	9.67 ± 0.58
*Staphylococcus oralis*	2/4	1/5	2/4	8.67 ± 0.58	8.33 ± 0.58	9.67 ± 0.58
*Staphylococcus pasteuri*	1/3	2/2	4/0	7.33 ± 0.58	6.67 ± 0.58	8.33 ± 0.58
*Staphylococcus warnerri*	1/2	0/3	1/2	9.33 ± 0.58	10.33 ± 0.58	11.33 ± 0.58
**Total R/S**	**16/49**	**24/41**	**24/41**			

Abbreviations: CAEO, *Cedar atlantica* essential oil; CIP, ciprofloxacin; IPM, imipenem; IVEO, *Illicium verum* essential oil; LZD, linezolid; ND, not detected; PGEO, *Pelargonium graveolens* essential oil; TOB, tobramycin; TGC, tigecycline; TE, tetracycline.

Table 5 shows the antibiotic resistance/susceptibility of the microbiota isolated from *Steatoda triangulosa*. A total of 77 species isolated from *S. triangulosa* showed resistance to various antibiotics while 160 isolates were sensitive to antibiotic treatment. CAEO showed the most potent antibacterial activity against *Priestia megaterium* and *Staphylococcus oralis* with an inhibition zone of 11.67 mm. *Lysinibacillus sphaericus* was the most sensitive bacterium to IVEO, while *Acinetobacter johnsonii* was the most resistant bacterium to PGEO.

**Table 5:** Antibiotic resistance and antibacterial activity of EOs against bacteria isolated from *Steatoda triangulosa*.

	Antibiotic resistance R/S			Antibacterial activity in mm		
Isolated bacteria	IMP	CIP	TOB	CAEO	IVEO	PGEO
*Acinetobacter johnsonii*	1/4	2/3	3/2	2.33 ± 0.58	2.67 ± 0.58	3.33 ± 0.58
*Aerococcus viridans*	ND	ND	ND	4.67 ± 0.58	5.67 ± 0.58	4.67 ± 0.58
	**IMP**	**CIP**	**LZD**	**CAEO**	**IVEO**	**PGEO**
*Bacillus altitudinis*	1/3	2/2	1/3	8.33 ± 0.58	6.67 ± 0.58	8.33 ± 0.58
*Bacillus cereus*	1/3	2/2	0/4	10.33 ± 0.58	9.33 ± 0.58	10.33 ± 0.58
*Priestia megaterium*	1/2	0/3	0/3	11.67 ± 0.58	10.33 ± 0.58	11.67 ± 0.58
*Bacillus mycoides*	2/4	3/3	2/4	7.67 ± 0.58	7.33 ± 0.58	7.67 ± 0.58
*Bacillus pumilus*	1/4	4/1	2/3	7.67 ± 0.58	6.67 ± 0.58	7.67 ± 0.58
*Bacillus thurgiensis*	1/3	0/4	2/2	6.67 ± 0.58	5.67 ± 0.58	7.33 ± 0.58
	**CIP**	**TET**	**LZD**	**CAEO**	**IVEO**	**PGEO**
*Corynebacterium singulare*	3/3	2/4	1/5	5.67 ± 0.58	4.33 ± 0.58	7.67 ± 0.58
*Corynebacterium xerosis*	2/5	3/4	1/6	8.33 ± 0.58	7.67 ± 0.58	8.67 ± 0.58
*Cupriavidus metallidurans*	ND	ND	ND	6.33 ± 0.58	10.33 ± 0.58	7.33 ± 0.58
	**CIP**	**TGC**	**LZD**	**CAEO**	**IVEO**	**PGEO**
*Enterococcus durans*	2/4	1/5	2/4	9.33 ± 0.58	10.67 ± 0.58	9.67 ± 0.58
*Enterococcus faecium*	4/3	5/2	1/6	8.33 ± 0.58	9.67 ± 0.58	8.33 ± 0.58
*Kocuria rhizophila*	ND	ND	ND	7.67 ± 0.58	7.33 ± 0.58	9.33 ± 0.58
	**IMP**	**CIP**	**LZD**	**CAEO**	**IVEO**	**PGEO**
*Lysinibacillus boronitolerans*	ND	ND	ND	9.67 ± 0.58	8.33 ± 0.58	8.67 ± 0.58
*Lysinibacillus fusiformis*	ND	ND	ND	9.67 ± 0.58	10.33 ± 0.58	11.33 ± 0.58
*Lysinibacillus sphaericus*	ND	ND	ND	8.67 ± 0.58	11.67 ± 0.58	10.33 ± 0.58
*Moraxella osloensis*	ND	ND	ND	8.67 ± 0.58	7.67 ± 0.58	9.33 ± 0.58
*Paenibacillus polymyxa*	ND	ND	ND	7.67 ± 0.58	7.67 ± 0.58	8.33 ± 0.58
*Stutzerimonas chloritidismutans*	ND	ND	ND	6.33 ± 0.58	7.33 ± 0.58	9.33 ± 0.58
*Roseomonas muscosa*	ND	ND	ND	8.67 ± 0.58	9.33 ± 0.58	10.67 ± 0.58
	**IMP**	**TOB**	**TGC**	**CAEO**	**IVEO**	**PGEO**
*Salmonella* spp.	1/3	2/2	0/4	9.33 ± 0.58	9.33 ± 0.58	8.67 ± 0.58
*Sphingomonas parapaucimobilis*	ND	ND	ND	8.33 ± 0.58	8.33 ± 0.58	7.33 ± 0.58
*Sphingomonas yabuuchiae*	ND	ND	ND	9.33 ± 0.58	9.33 ± 0.58	8.67 ± 0.58
	**TOB**	**TGC**	**LZD**	**CAEO**	**IVEO**	**PGEO**
*Staphylococcus epidermidis*	1/2	2/1	0/3	8.33 ± 0.58	9.33 ± 0.58	8.33 ± 0.58
*Staphylococcus hominis*	2/3	1/4	0/5	10.33 ± 0.58	8.33 ± 0.58	9.67 ± 0.58
*Staphylococcus oralis*	0/3	1/2	0/3	11.67 ± 0.58	9.33 ± 0.58	9.33 ± 0.58
*Staphylococcus pasteuri*	1/2	2/1	3/0	7.67 ± 0.58	7.67 ± 0.58	8.33 ± 0.58
*Staphylococcus warnerri*	2/2	1/3	0/4	7.67 ± 0.58	8.33 ± 0.58	9.33 ± 0.58
**Total R/S**	**26/53**	**33/46**	**18/61**			

Abbreviations: CAEO, *Cedar atlantica* essential oil; CIP, ciprofloxacin; IPM, imipenem; IVEO, *Illicium verum* essential oil; LZD, linezolid; ND, not detected; PGEO, *Pelargonium graveolens* essential oil; TOB, tobramycin; TGC, tigecycline; TE, tetracycline.

Antibiotic resistance/sensitivity of microbiota isolated from *Tegenaria domestica* is shown in Table 6. In total, 96 species isolated from *T. domestica* were resistant to different antibiotics. Sensitivity to antibiotic resistance was found in 123 isolates. The best antibacterial activity of CAEO was found against *Bacillus pumilus, Enterococcus durans* and *Staphylococcus pasteuri* (12.33 mm). The most resistant bacterium against IVEO was *Sphingomonas parapaucimobilis* (3.33 mm) and most sensitive bacterium against PGEO was *Bacillus thurgiensis* (14.67 mm).

**Table 6:** Antibiotic resistance and antibacterial activity of EOs against bacteria isolated from *Tegenaria domestica*.

	Antibiotic resistance R/S			Antibacterial activity in mm		
Isolated bacteria	IMP	CIP	TOB	CAEO	IVEO	PGEO
*Acinetobacter johnsonii*	3/2	1/4	0/5	7.67 ± 0.58	8.33 ± 0.58	7.67 ± 0.58
*Aerococcus viridans*	ND	ND	ND	8.33 ± 0.58	9.67 ± 0.58	8.67 ± 0.58
	**IMP**	**CIP**	**LZD**	**CAEO**	**IVEO**	**PGEO**
*Bacillus altitudinis*	2/2	1/3	3/1	7.33 ± 0.58	6.67 ± 0.58	8.67 ± 0.58
*Bacillus cereus*	3/0	1/2	2/1	6.67 ± 0.58	7.33 ± 0.58	8.33 ± 0.58
*Priestia megaterium*	2/3	3/2	1/4	7.33 ± 0.58	4.67 ± 0.58	6.67 ± 0.58
*Bacillus mycoides*	2/4	3/3	4/2	4.67 ± 0.58	12.33 ± 0.58	9.67 ± 0.58
*Bacillus pumilus*	2/3	3/2	1/4	12.33 ± 0.58	11.67 ± 0.58	8.67 ± 0.58
*Bacillus thurgiensis*	2/2	1/3	4/0	11.67 ± 0.58	13.33 ± 0.58	14.67 ± 0.58
	**CIP**	**TET**	**LZD**	**CAEO**	**IVEO**	**PGEO**
*Corynebacterium singulare*	1/3	4/0	1/3	6.67 ± 0.58	7.33 ± 0.58	7.67 ± 0.58
*Corynebacterium xerosis*	1/2	0/3	0/3	8.33 ± 0.58	11.33 ± 0.58	9.67 ± 0.58
*Cupriavidus metallidurans*	ND	ND	ND	8.67 ± 0.58	9.33 ± 0.58	5.67 ± 0.58
	**CIP**	**TGC**	**LZD**	**CAEO**	**IVEO**	**PGEO**
*Enterococcus durans*	1/4	2/3	4/1	12.33 ± 0.58	13.33 ± 0.58	12.67 ± 0.58
*Enterococcus faecium*	2/4	3/3	2/4	11.67 ± 0.58	10.67 ± 0.58	9.33 ± 0.58
*Kocuria rhizophila*	ND	ND	ND	6.67 ± 0.58	4.33 ± 0.58	7.33 ± 0.58
	**IMP**	**CIP**	**LZD**	**CAEO**	**IVEO**	**PGEO**
*Lysinibacillus boronitolerans*	ND	ND	ND	6.67 ± 0.58	5.33 ± 0.58	7.33 ± 0.58
*Lysinibacillus fusiformis*	ND	ND	ND	7.67 ± 0.58	6.67 ± 0.58	5.67 ± 0.58
*Stutzerimonas chloritidismutans*	ND	ND	ND	8.33 ± 0.58	7.67 ± 0.58	9.33 ± 0.58
*Roseomonas muscosa*	ND	ND	ND	5.67 ± 0.58	4.33 ± 0.58	6.33 ± 0.58
	**IMP**	**TOB**	**PIP**	**CAEO**	**IVEO**	**PGEO**
*Salmonella* spp.	1/2	3/0	0/3	7.67 ± 0.58	8.33 ± 0.58	11.67 ± 0.58
*Sphingomonas parapaucimobilis*	ND	ND	ND	4.67 ± 0.58	3.33 ± 0.58	5.33 ± 0.58
*Sphingomonas yabuuchiae*	ND	ND	ND	6.67 ± 0.58	7.33 ± 0.58	8.33 ± 0.58
	**TOB**	**TGC**	**LZD**	**CAEO**	**IVEO**	**PGEO**
*Staphylococcus epidermidis*	1/3	2/2	4/0	6.67 ± 0.58	7.33 ± 0.58	8.67 ± 0.58
*Staphylococcus hominis*	1/2	0/3	3/0	7.33 ± 0.58	9.67 ± 0.58	11.33 ± 0.58
*Staphylococcus oralis*	2/2	4/0	3/1	4.67 ± 0.58	7.33 ± 0.58	4.67 ± 0.58
*Staphylococcus pasteuri*	2/2	1/3	0/4	12.33 ± 0.58	13.33 ± 0.58	8.67 ± 0.58
*Staphylococcus warnerri*	3/2	1/4	0/5	11.67 ± 0.58	12.67 ± 0.58	10.67 ± 0.58
**Total R/S**	**31/42**	**33/40**	**32/41**			

Abbreviations: CAEO, *Cedar atlantica* essential oil; CIP, ciprofloxacin; IPM, imipenem; IVEO, *Illicium verum* essential oil; LZD, linezolid; ND, not detected; PGEO, *Pelargonium graveolens* essential oil; TOB, tobramycin; TGC, tigecycline; TE, tetracycline.

## 4 Discussion

Recent studies have highlighted the significant role of arthropod microbiota in the transmission of animal infections, human health, and the dissemination of antibiotic resistance genes (Schapheer et al. 2021). Reports have confirmed the crucial role of insects in maintaining pathogens and antibiotic resistance genes within agricultural environments, particularly in livestock and poultry production premises where potentially virulent, antimicrobial‐resistant enterococci have been identified (Gwenzi et al. 2021). This underscores the importance of researching arthropod microbiota, especially in highly contaminated environments like poultry farms with high bird stocking densities (Alig et al. 2023). In our study, microbial counts in spider samples ranged from 1.21 log CFU/g to 2.02 log CFU/g across different samples and media types. These findings are consistent with the results reported by Kačániová et al. (2022b), which showed comparable microbial counts across different spider species and habitats.

Previous research has investigated the bacterial communities within specific spider species (Vanthournout and Hendrickx 2015; Zhang et al. 2017). Zhang et al. (2018), examined bacterial populations across eight spider species and found diverse bacterial communities and endosymbionts within their bodies. They also studied the distribution and relative abundance of bacteria and endosymbionts among different spider hosts. Their findings indicated variability in symbiont distribution across hosts, with some spiders exhibiting higher abundance of specific bacteria such as *Rhizobium*, *Methylobacterium*, *Brevundimonas*, and *Sphingomonas*, notably observed in *Dictis striatipes* compared to other species. In our investigation, we isolated 29 species, 16 genera, and 13 families from *Pardosa hortensis* spiders. The most frequently isolated species included *Bacillus mycoides*, *Bacillus thurgiensis*, *Sphingomonas parapaucimobilis*, *Staphylococcus warneri*, and *Stutzerimonas chloritidismutans*. Vanthournout and Hendrickx (2015), studied the bacterial communities of *Oedothorax gibbosus*, highlighting the prevalence of endosymbionts with no other bacteria identified. Zhang et al. (2017) identified bacterial communities in *Marpiss magister* spiders, noting the presence of various bacteria alongside endosymbionts. Furthermore, Zhang et al. (2018), expanded their study to include additional bacteria beyond endosymbionts, such as *Pseudomonas*, *Sphingomonas*, *Acinetobacter*, *Novosphingobium*, *Aquabacterium*, *Methylobacterium*, *Brevundimonas*, *Rhizobium*, *Bradyrhizobium*, *Arthrobacter*, *Pseudonocardia*, *Microbacterium*, *Citrobacter*, *Lactobacillus*, and *Lactococcus*, across spider communities. In *P. phalangioides*, *Bacillus mycoides*, *Staphylococcus oralis*, and *Staphylococcus pasteuri* were frequently identified. *Steatoda bipunctata* spiders harbored 27 species, with *Stutzerimonas chloritidismutans* being the predominant isolate. *Steatoda triangulosa* spiders yielded 29 isolated species, with notable isolates including *Aerococcus viridans*, *Corynebacterium singulare*, *Corynebacterium xerosis*, *Enterococcus durans*, *Enterococcus faecium*, and *Sphingomonas parapaucimobilis*. Among *Tegenaria domestica* spiders, 26 species were identified, spanning 14 genera and 12 families, with *Enterococcus faecium* and *Bacillus mycoides* being the most prevalent species. Our findings indicated consistent bacterial and endosymbiont abundances across various spider species. In study Kačániová et al. (2022b), spiders analyzed in our research harbored *Bacillus*, *Paenibacillus*, *Pseudomonas*, and *Staphylococcus* spp., with *Bacillus thurgiensis* present universally. Notably, *B. thurgiensis*, a soil‐dwelling microorganism known for its insecticidal properties and occasional human infection cases, along with established human pathogens like *Klebsiella pneumoniae*, *Escherichia coli*, and *Salmonella* spp., were identified. These results corroborate our findings, highlighting the presence of diverse bacterial communities and pathogens within spider populations, underscoring their ecological and potentially public health significance.

In our study, 93 different species isolated from *Pardosa hortensis* showed resistance to various antibiotics. Similarly, 93 distinct species from *Pholcus phalangioides* exhibited antibiotic resistance. Additionally, 64 species from *Steatoda bipunctata* and 77 species from *Steatoda triangulosa* demonstrated resistance to antibiotics. Furthermore, 96 species isolated from *Tegenaria domestica* were found to be resistant to antibiotics. The antibacterial resistance of spider's surface microbiomes was evident across all sampled sites in the study by Kačániová et al. (2022b), with the highest prevalence observed in slaughterhouses (38%), followed by buildings (23%) and chicken farms (7%). Arthropods, such as spiders, are numerous animals that interact extensively with their environments (Gonçalves and Pereira 2012). They harbor diverse microbial populations, including both Gram‐positive (*Enterococcus* spp., *Staphylococcus* spp.) and Gram‐negative (*Enterobacter* spp., *Klebsiella* spp.) bacteria resistant to antibiotics (Odoi et al. 2020). Synanthropic arthropods act as vectors for spreading antimicrobial‐resistant (AMR) bacteria in various environments. For example, houseflies have been implicated in the dissemination of Enterobacteriaceae resistant to cephalosporins and colistin (CST) (Fukuda et al. 2018). Another study identified multidrug‐resistant Enterobacteriaceae from cockroaches. Although humans and animals harbor large populations of Enterobacteriaceae in their digestive systems, little research has explored the role of other wild arthropods in AMR bacterial transmission (Odoi et al. 2020).

Acinetobacter johnsonii, Bacillus altitudinis, Moraxella osloensis, Priestia megaterium, *Staphylococcus epidermidis*, Staphylococcus oralis, and Staphylococcus warnerii were effectively inhibited by CAEO. IVEO exhibited the strongest antibacterial activity against Staphylococcus hominis, Lysinibacillus sphaericus, and Moraxella osloensis. PGEO showed the most effective antibacterial efficacy against Bacillus thurgiensis, *Staphylococcus epidermidis*, and Moraxella osloensis. Previous studies have indicated that CAEO has potent antibacterial activity against Bacillus subtilis, Bacillus cereus, and *Escherichia coli* (Zrira and Ghanmi 2016). Derwich et al. (2010), reported that C. atlantica leaf oil exhibits moderate antibacterial activity against Enterococcus faecalis and Pseudomonas aeruginosa. Similarly, Satrani et al. (2006), found antibacterial action of C. atlantica essential oil against B. subtilis, *E. coli*, Micrococcus luteus, and *Staphylococcus aureus*. Rhafouri et al. (2014), demonstrated antibacterial activity against *Staphylococcus aureus* and Enterococcus faecalis using essential oils extracted from cedar seeds. The antibacterial potential of IVEO against methicillin‐resistant *Staphylococcus aureus* (MRSA) clinical isolates has been demonstrated in multiple studies (Maqbul et al. 2019; Maqbul et al. 2020a; Maqbul et al. 2020b). Studies by (Kačániová et al. 2024b), validated the antibacterial activity of IVEO against biofilm‐forming Salmonella enterica and both Gram‐positive and Gram‐negative bacteria using disk diffusion and microdilution methods. Gram‐positive bacteria have been shown to be more susceptible to the effects of essential oils compared to Gram‐negative bacteria (Shan et al. 2007; Thirumurugan et al. 2010). Freire et al. (2011), demonstrated the effectiveness of IVEO against *E. coli* strains. Noumi et al. (2023), also found superior efficacy of IVEO against *Staphylococcus aureus* compared to Pseudomonas aeruginosa, Shigella flexneri, and Vibrio vulnificus. Damayanti et al. (2019), reported that IVEO exhibited greater efficacy against *S. aureus* than *E. coli* using the disc diffusion method. Moreover, Muhsinah et al. (2022), highlighted the effectiveness of IVEO against methicillin‐resistant *Staphylococcus aureus* (MRSA) strains. Except for Listeria monocytogenes, all bacteria tested showed susceptibility to PGEO, and susceptibility varied with oil dilution. Ghannadi et al. (2012), reported that neat PGEO demonstrated the largest inhibitory zones compared to amoxicillin and chloramphenicol, especially against Gram‐positive bacteria. Kačániová et al. (2023a), also found that Gram‐positive strains exhibited higher sensitivity to PGEO, with notable activity against biofilm‐forming Gram‐negative bacteria Salmonella enterica. Al‐Mijalli et al. 2022), demonstrated potent antibacterial effects of PGEO against all tested strains except Salmonella typhimurium. Hsouna and Hamdi (2012), highlighted the strong inhibitory activity of PGEO against various bacterial species, particularly Gram‐positive species such as Bacillus cereus ATCC 14579 and *Staphylococcus aureus* ATCC 25923. The potential application of essential oils as antimicrobial agents is promising based on their demonstrated antibacterial activities. Further research is needed to determine optimal concentrations and the mechanism of action for these essential oils. Additionally, more studies are necessary to confirm their safety and identify the primary antibacterial components (Angane et al. 2022). Compared to Gram‐positive bacteria, Gram‐negative bacteria often exhibit greater resistance to EOs (Trombetta et al. 2005). Because of the way the cell wall of Gram‐positive bacteria is structured, hydrophobic compounds can readily enter the cell and affect the cytoplasm as well as the cell wall. The EOs also contain phenolic chemicals, which often have antibacterial activity against Gram‐positive bacteria (Tiwari et al. 2009). The chemical composition of EOs and/or their constituents determines their modes of action (Dorman and Deans 2000). One or more objectives may be the focus of the action of EOs and/or its constituents (Helander et al. 1998, 1997).

## 5 Conclusions

Our study focused on spider samples primarily collected from the same habitat, thereby reducing the likelihood of bacterial contamination affecting variations in bacterial abundance. Spiders are among the most diverse and prevalent predators in agroecosystems, harboring a diverse microbiota on their external surfaces, predominantly from the phylum Proteobacteria, with *Bacillus* and *Staphylococcus* being the most prevalent genera. Our research revealed opportunistic pathogenic bacteria on the body surfaces of five spider species. These bacteria have the potential to disseminate various diseases, including zoonoses, through vector‐borne transmission. We identified spider isolates that potentially contribute to the spread of AMR, demonstrating multidrug resistance and resistance to antimicrobials critical for human therapy. This study expands our understanding of AMR profiles in microbial isolates and their compositions, with implications for environmental and public health. Our findings underscore the potential of essential oils as effective alternatives to traditional methods for managing antibiotic‐resistant bacteria. Furthermore, they emphasize the significance of EOs in advancing novel antimicrobial strategies beyond conventional therapeutic approaches.

## Author Contributions

**Miroslava Kačániová:** writing – review and editing, writing – original draft, project administration, supervision, formal analysis, investigation, methodology, conceptualization, data curation, funding acquisition, resources. **Joel Horacio Elizondo‑Luevano:** writing – review and editing, writing – original draft, methodology, formal analysis. **Zhaojun Ban:** writing – review and editing, writing – original draft, methodology, formal analysis. **Mária Babošová:** writing – review and editing, writing – original draft, methodology, formal analysis. **Jana Ivanič Porhajašová:** writing – review and editing, writing – original draft, methodology, formal analysis. **Ján Kollár:** writing – review and editing, writing – original draft, methodology, formal analysis. **Przemysław Łukasz Kowalczewski:** writing – review and editing, writing – original draft, methodology, formal analysis. **Stefania Garzoli:** writing – review and editing, writing – original draft, methodology, conceptualization, visualization, data curation. All authors have read and agreed to the published version of the manuscript.

## Ethics Statement

The authors have nothing to report.

## Conflicts of Interest

The authors declare no conflicts of interest.

## Data Availability

All data analyzed during this study are included in the published article. Further inquiries can be directed to the corresponding author.
